# Supplementary material for: Developmental progression of DNA double-strand break repair deciphered by a single-allele resolution mutation classifier
Source: Nat Commun. 2024 Mar 23;15:2629. doi: 10.1038/s41467-024-46479-2 (PMC10960810; doi:10.1038/s41467-024-46479-2)
Supplement: Supplementary file 1 — Supplementary Information [file 41467_2024_46479_MOESM1_ESM.pdf]

Supplementary Information

Developmental progression of DNA double-strand break repair deciphered by a new single-allele resolution mutation classifier

Zhiqian Li[1,2], Lang You[1,2], Anita Hermann[1,2], Ethan Bier[1,2*]

[1] Department of Cell and Developmental Biology, University of California, San Diego, La Jolla, CA 92093, USA
[2] Tata Institute for Genetics and Society, University of California, San Diego, La Jolla, CA 92093, USA

* Correspondence: ebier@ucsd.edu

Supplementary Discussion

New features of ICPs

The new ICP DSB classifier pipeline provides a substantial analytic advance with the following features: 1) creating comprehensive sequence dictionaries for the major mutation categories including PEPPR, MMEJ, and non-characterized deletions (DELET), enabling single allele resolution analysis; 2) efficient categorization of DSBs using the SAClassifier, which requires only ~5 minutes/experiment of investigator effort (while the knock-knock pipeline that Hussmann *et al*. used in their paper employs a fragmented local alignment, which complicates iterative alignment and often causes errors during reconstructing the reads); 3) semi-automation: no manual inspection and correction are needed once the dictionaries have been properly built since the alleles derive from real reads of sequencing data. These combined features substantially streamlined analysis of editing outcomes from the maternal crosses with the *ple* and Rec*kh* Cas9/gRNAs since in both of these cases very dominant somatic indels were created necessitating in-depth views of lower frequency editing outcomes; 4) development of clean 24-nt sub-dictionaries to aid in error calling to refine the SAClassifier instead of full-length dictionaries that have lower correction power; 5) numerical tagging of single alleles, enabling facile single allele tracking in diverse experimental contexts; 6) validated resolution of seemingly ambiguous and/or complex alleles that span multiple classes (e.g., some PEPPR alleles were precisely flanked by microhomologies at the cutting site, which here we defined as MMEJ rather than PEPPR and assigning complex "indel" alleles comprising both deletions and insertions to specific insertion events); and 7) a more informative and intuitive outputs displayed the classification outcomes by rank-ordered and color-coded DSB repair fingerprints, which conveying the information of allele frequency, class, as well as the overall DSB repair patterns which is especially being meaningful when compared different crosses or origin of Cas9 sources. These integrated components of our new ICP pipeline rendered through a novel intuitive graphical interface enables comprehensive interpretation and comparisons of diverse DSB repair outcomes.

ICPs uncover the contribution of target gene fitness to somatic DSB repair patterns

We also observed distinctive somatic DSB repair signatures for cutting at the *Spo11*, *prosα2*, *Rab5* and *Rab11* loci in flies, with *Spo11* gRNA displaying the strongest distinguishable DSB repair signatures between maternal versus paternal crosses. These distinctions were not as striking for the *Rab5* and *prosα2* gRNAs and yet less so in the case of the *Rab11* gRNA, indicating that these latter three scenarios resulted in more blended outcomes based on parental genotype. While we are uncertain what the basis for these locus-specific differences are, we speculate that the *Drosophila* gRNAs manifesting weak parental inheritance signatures were obtained with recorded cDNA sequences (rescuing the interrupted target gene) inserted into loci required for viability of the organism. It is possible that heterozygosity or mosaic partial homozygosity for loss-of-function alleles may be associated with significant fitness costs that could result in such alleles being culled over development of the individual. Similar purifying forms of selection have been observed in transgenerational experiments in which individuals carrying both a source of Cas9 and gRNA in the presence of WT target alleles manifest reduced fitness in a process we have referred to as lethal mosaicism. This effect would be expected to be less intense for the *Spo11* gRNA where non-functional NHEJ alleles result in sterility rather than lethality consistent with the *Spo11* gRNA providing strong sex-specific DSB signatures.

As discussed further below, however, the *An. stephensi* Rec*kh* gRNA did generate very strong sex-specific ICP profiles, indicating that drives carrying a recoded transgene do not necessarily erase such allelic differences in all cases. Further studies will be required to define the key parameters permitting maximal discrimination of DSB outcomes using the ICP platform.

A potential mutant allele selection revealed by time-course experiment

One curious observation in our time-course data was that following a very early generation of mutant alleles (within 30 minutes after fertilization), we observed a rebound in the proportion of unedited WT alleles. While this counterintuitive finding may result from stochastic sampling of populations of embryos (20 embryos per sample), we observed similar trajectories at fly *prosα2* gRNA target (Fig. S8a). Also, we note that these variations were fairly large to be accounted for by sampling bias alone. One potential explanation for this surprising transient resurgence of WT alleles is that cells carrying mutant alleles may experience fitness costs that reduce their ability to generate clonal progeny, in line with the hypothesis outlined above regarding the potential role of somatic cell fitness in determining the DSB fingerprints.

Supplementary Table 1: gRNA targeting sequences

Target	Sequences (5'-3')
Dmple	GAAGTCCGTGTTAGTGACACTGG
DmRab5	CACTTGCAGCAGTTGTTCGTCGG
DmRab11	GCACTGTTTGCGCACATCGGCGG
DmSpo11	GATTGTCATAGTATAGTCCACGG
Dmprosα2	CGTCCAACTGGAGTATGCATTGG
Askh	GATGGTTCCGTTCTACGGGCAGG
Dmhth	GCATGCACGGCTACCATTCGGGG

Supplementary Table 2: Primers used in this study

Name	Primer sequence (5'-3')
Rab5-NGSF	ACACTCTTTCCCTACACGACGCTCTTCCGATCTGGCGCCAACAATCAGGGAAC
Rab5-NGSR	GTGACTGGAGTTCAGACGTGTGCTCTTCCGATCTCTGTGTTAGTAATGCAACCGTATT
Rab11-NGSF	ACACTCTTTCCCTACACGACGCTCTTCCGATCTGGAAGGCGACGTCATCCG
Rab11-NGSR	GTGACTGGAGTTCAGACGTGTGCTCTTCCGATCTGCTGTTGCTTCTGTTTATGTTG
Spo11-NGSF	ACACTCTTTCCCTACACGACGCTCTTCCGATCtGCTGATTTACATGCTCTCCCG
Spo11-NGSR	GTGACTGGAGTTCAGACGTGTGCTCTTCCGATCTCCTCAGCATACGACAGACATC
prosα2-NGSF	ACACTCTTTCCCTACACGACGCTCTTCCGATCTCCGCCATGACTGGGCAATAAGT
prosα2-NGSR	GTGACTGGAGTTCAGACGTGTGCTCTTCCGATCTGCTGTGCGGCATGCTAACCTATAAT
Kh-NGSF	ACACTCTTTCCCTACACGACGCTCTTCCGATCTGTACCACGTGGGATCGAAGGC
Kh-NGSR	GACTGGAGTTCAGACGTGTGCTCTTCCGATCTCCGTACCGTACTGGTTGAACAG
pleCC-NGSF	ACACTCTTTCCCTACACGACGCTCTTCCGATCTGAAGAAAGCCACCCTGGCTTGA
pleCC-NGSR	GACTGGAGTTCAGACGTGTGCTCTTCCGATCTTCTCGAAACGGGCATCATCCACCAG
pleHDRRound1F	GTCGGACATCTCCTGCAACT
pleHDRRound1R	CGAGGGTTCGAAATCGATAA
pleHDR-NGSF	ACACTCTTTCCCTACACGACGCTCTTCCGATCTGGAGTAGTCGATGAATGGGATCGG
pleHDR-NGSR	GACTGGAGTTCAGACGTGTGCTCTTCCGATCTCGATTGTTTAGCTTGTTCAGCTGCG
hthHDR-Round1F	CCTTTGTCTTGCCTTTTCCACAAT
hthHDR-Round1R	CGAGGGTTCGAAATCGATAA

hthHDR-NGSF	ACACTCTTTCCCTACACGACGCTCTTCCGATCTAGTTGCAACACATGAAAATTG
hthHDR-NGSR	GACTGGAGTTCAGACGTGTGCTCTTCCGATCTCTCGCTCTGCGGTTCGGGTGTTA

Supplementary Figures

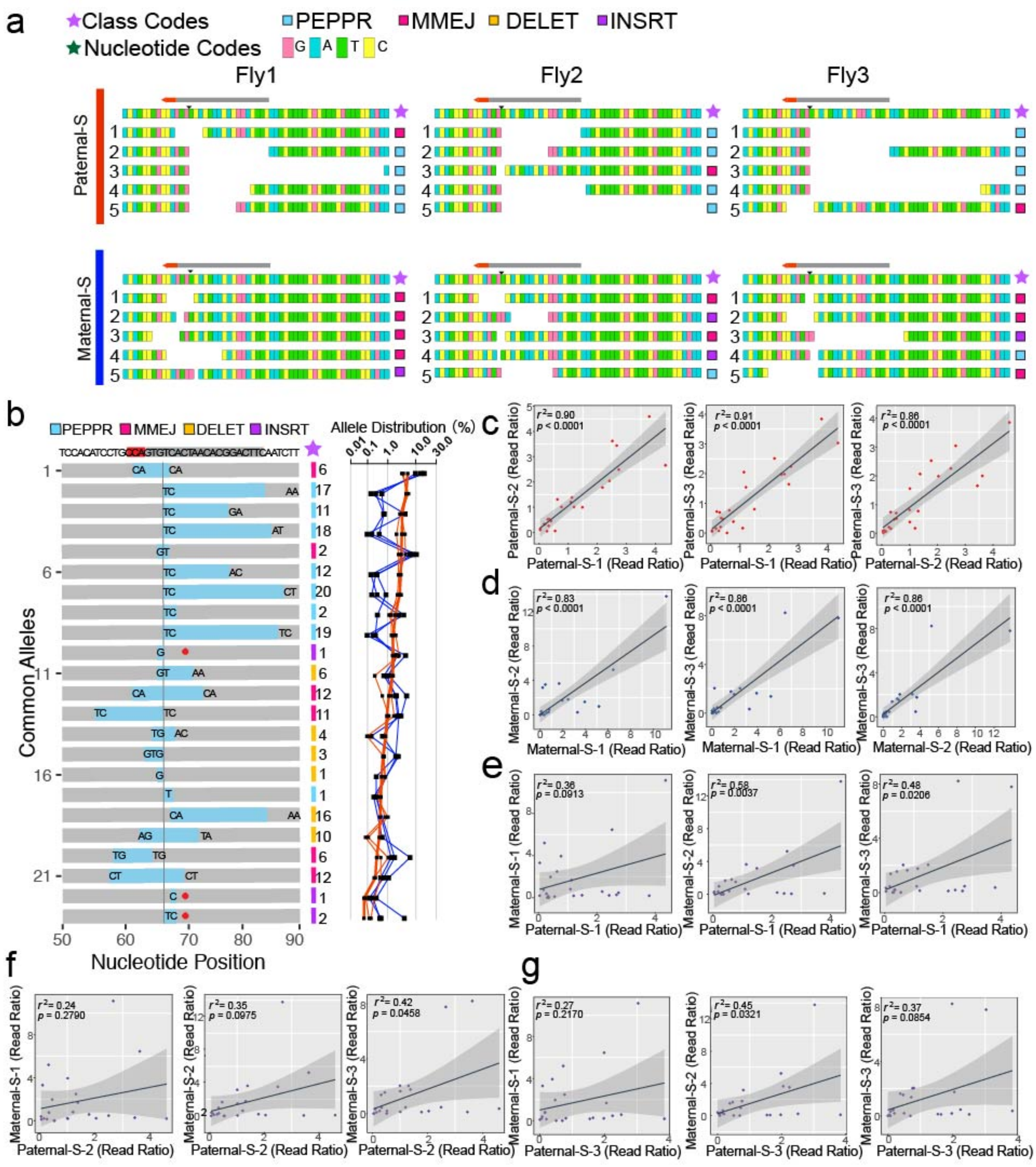

Suppl Figure 1. **Somatic DSB repair outcomes generated by *ple* gRNA and *vasa*-Cas9. a**. Top five somatic indels from three individually sequenced flies with Paternal-S (red rectangle) or Maternal-S (dark blue rectangle)

crosses. Top rows show the reference sequences with color coded nucleotides and expected gRNA cutting site indicated with black arrowhead. Gray rectangles indicate the gRNA binding site and red arrowheads are PAM sequences. Right color-coded squares indicate categories for each allele. **b**. 23 common alleles extracted from six sequenced single flies. The plot shows a 40 bp reference sequence window centered by the expected gRNA cutting site. Gray rectangle on the top row marks the protospacer and PAM is marked with a red rectangle. Vertical gray line shows the expected DSB cutting site. Gray rectangles below the reference sequence are targeted genomic DNA loci, blue rectangles show editing windows. Insertion alleles are marked with pink diamonds. The right side shows allele categories. The right plot shows the landscape of 23 common alleles ordered by one Paternal-S sample indicated by a bold red line. Reads ratio is displayed by probability (%) with GraphPad. **c-g.** Correlation analysis between two replicates within the Paternal-S cross (c), Maternal-S (d) or different combinations between Paternal-S and Maternal-S (e, f, g). Source data for b-g are provided as a Source Data file. r^2 values and p-values are indicated.

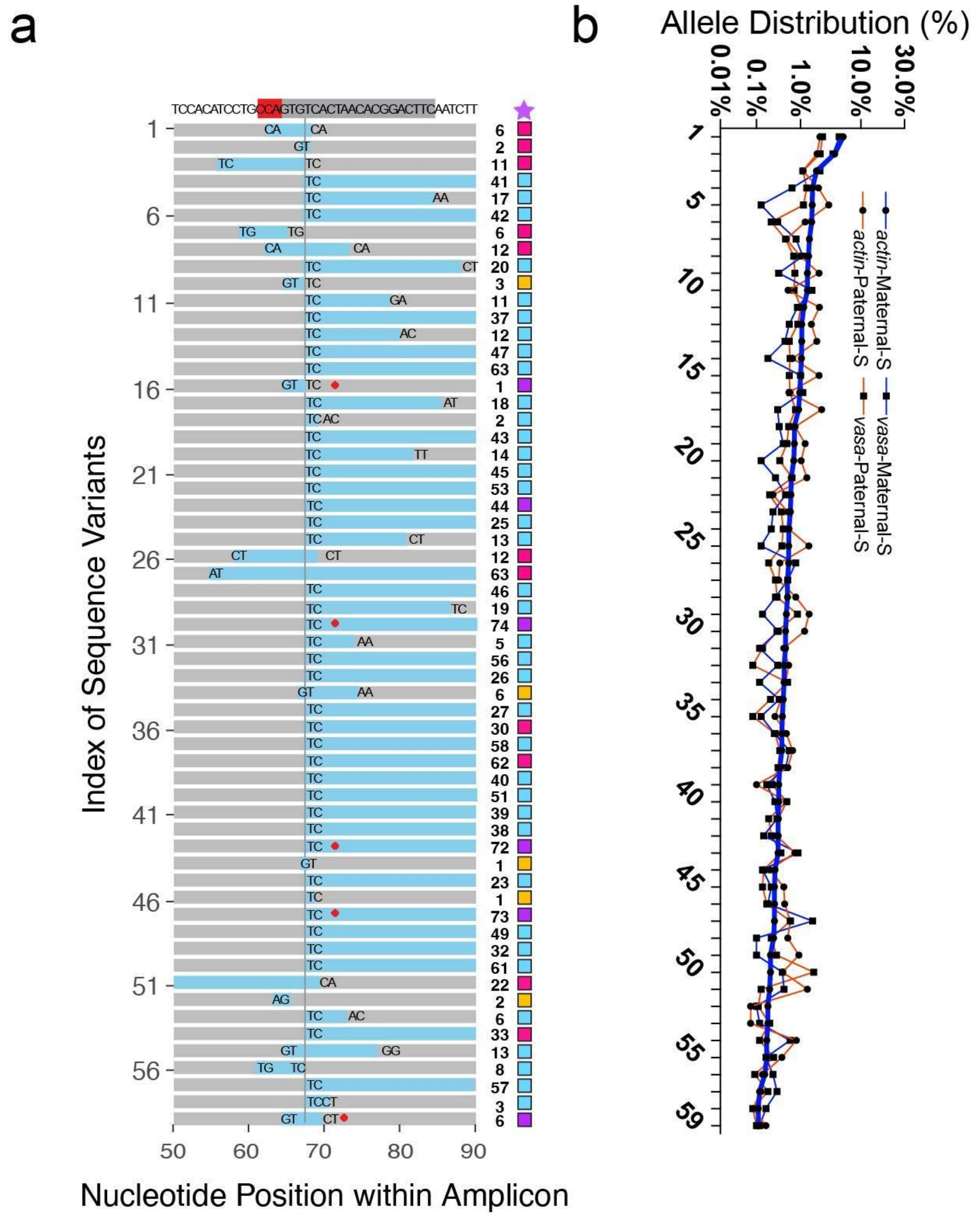

Suppl Figure 2. **Common alleles from *ple* gRNA crossed with *actin*-Cas9 and *vasa*-Cas9. a**. Common 59 alleles extracted from the *actin*-Cas9 and *vasa*-Cas9 crosses. The first row shows the reference sequence centered by the expected DSB cleavage site. gRNA binding site is labeled with gray rectangle and PAM sequence is red rectangle. Alleles are ranked by Maternal-S cross with *actin*-Cas9. Blue bars show the editing window which was marked with two nucleotides located on the editing boundaries. The right-side numbers indicate deletion size and colored squares are categories each allele belongs to. **b**. Landscape of the common 59 alleles with *actin*-Cas9 and *vasa*-Cas9 crosses. Dark blue line with round dots: Maternal-S cross with *actin*-Cas9, dark blue line with square dots: Maternal-S cross

with *vasa*-Cas9, red line with round dots: Paternal-S cross with *actin*-Cas9, red line with square dots is Paternal-S cross with *vasa*-Cas9. Fraction of each allele is shown with probability. Y-axis is the allele.

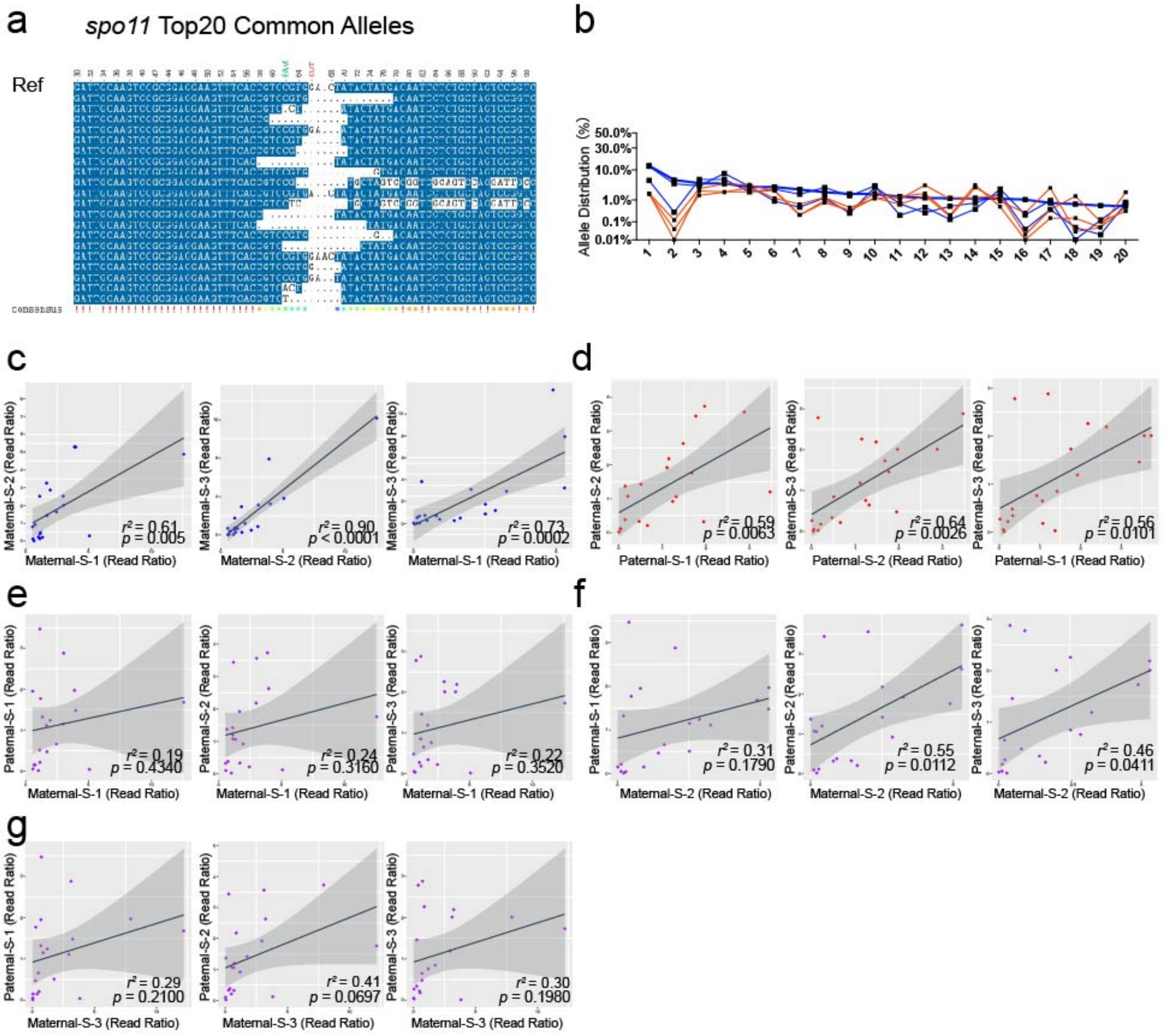

Suppl Figure 3. Correlation analysis for somatic DSB repair outcomes generated by *Spo11* gRNA. a. Top 20 common alleles across all six sequenced individual flies aligned with multiple sequences alignment (MSA) method. The first row is reference genome DNA sequence. Blue letters show consensus. **b**. Allele landscapes of top 20 common alleles. Dark blue lines show the alleles landscape from Maternal-S crosses and red lines are Paternal-S crosses. X-axis shows the top 20 alleles ranked by alleles frequency, y-axis is the alleles fraction showing the probability. **c-g**. Correlation analysis either between two samples within the Maternal-S cross (c), Paternal-S (d), or different combinations between two crosses (e, f, g). Source data for c-g are provided as a Source Data file. r^2 values and p-values are indicated.

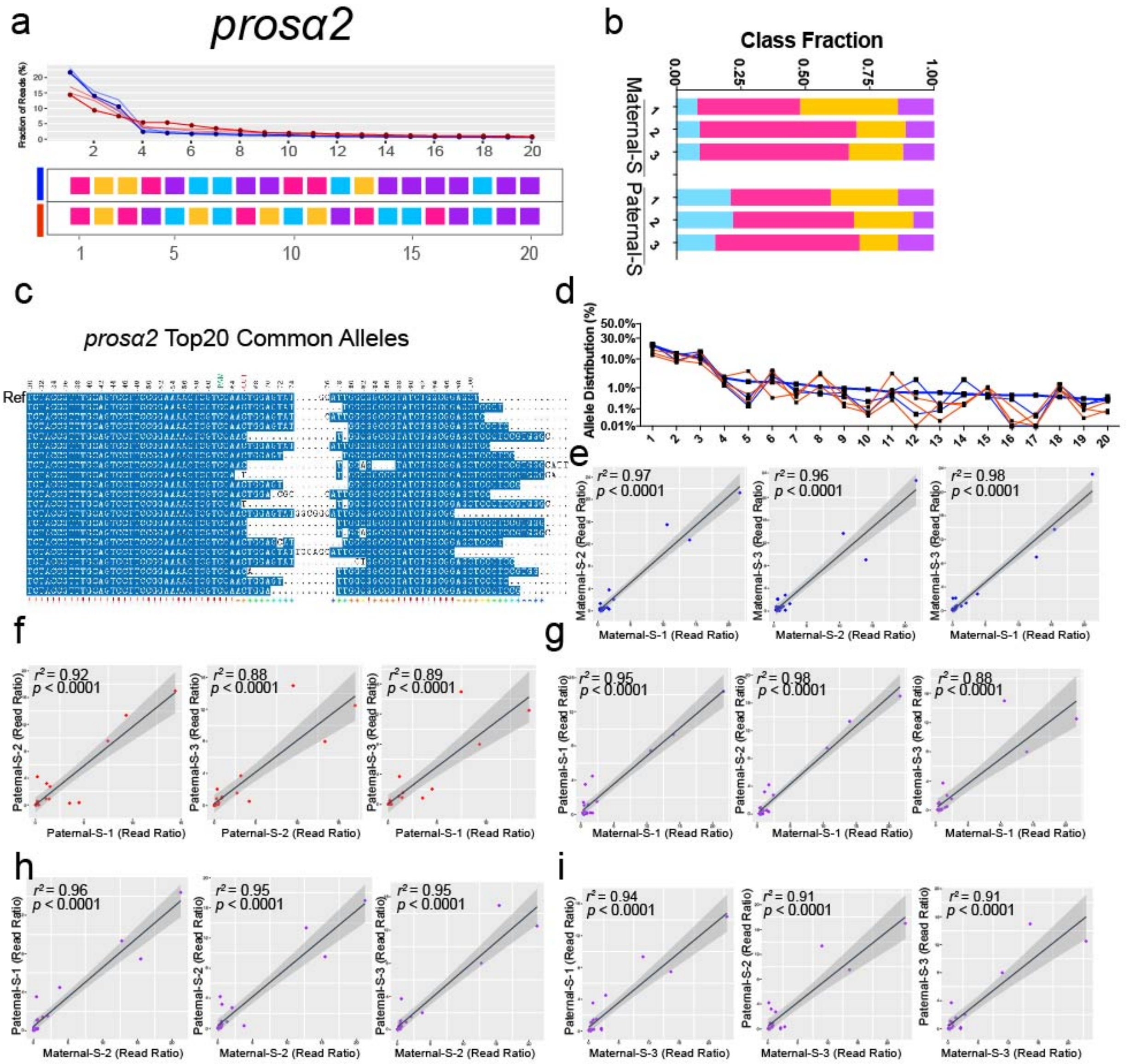

Suppl Figure 4. ICP analysis for somatic DSB repair outcomes generated by *prosα2* gRNA. a. Allele landscapes and classification fingerprints of top 20 alleles from *prosα2* Maternal-S (dark blue) and Paternal-S (red) crosses. For classification fingerprints, each allele is color coded with the allele categories. **b**. Class Fraction Index for top 50 alleles. **c**. Top 20 common alleles across all six sequenced individual flies aligned by multiple sequences alignment (MSA) method. The first row is the reference sequence. Blue letters show consensus among all aligned alleles. **d**. Allele landscapes of top 20 common alleles across all six sequenced flies. X-axis shows the top 20 alleles ranked by frequency, and the y-axis is the fraction of each allele showing the probability. Dark blue lines are alleles generated from Maternal-S crosses and red lines indicate Paternal-S crosses. **e-i**. Correlation analysis either between two samples within the Maternal-S cross (e), Paternal-S (f), or different combinations between two crosses (g, h, i). Source data for panels e-i are provided as a Source Data file. r^2 values and p-values are indicated.

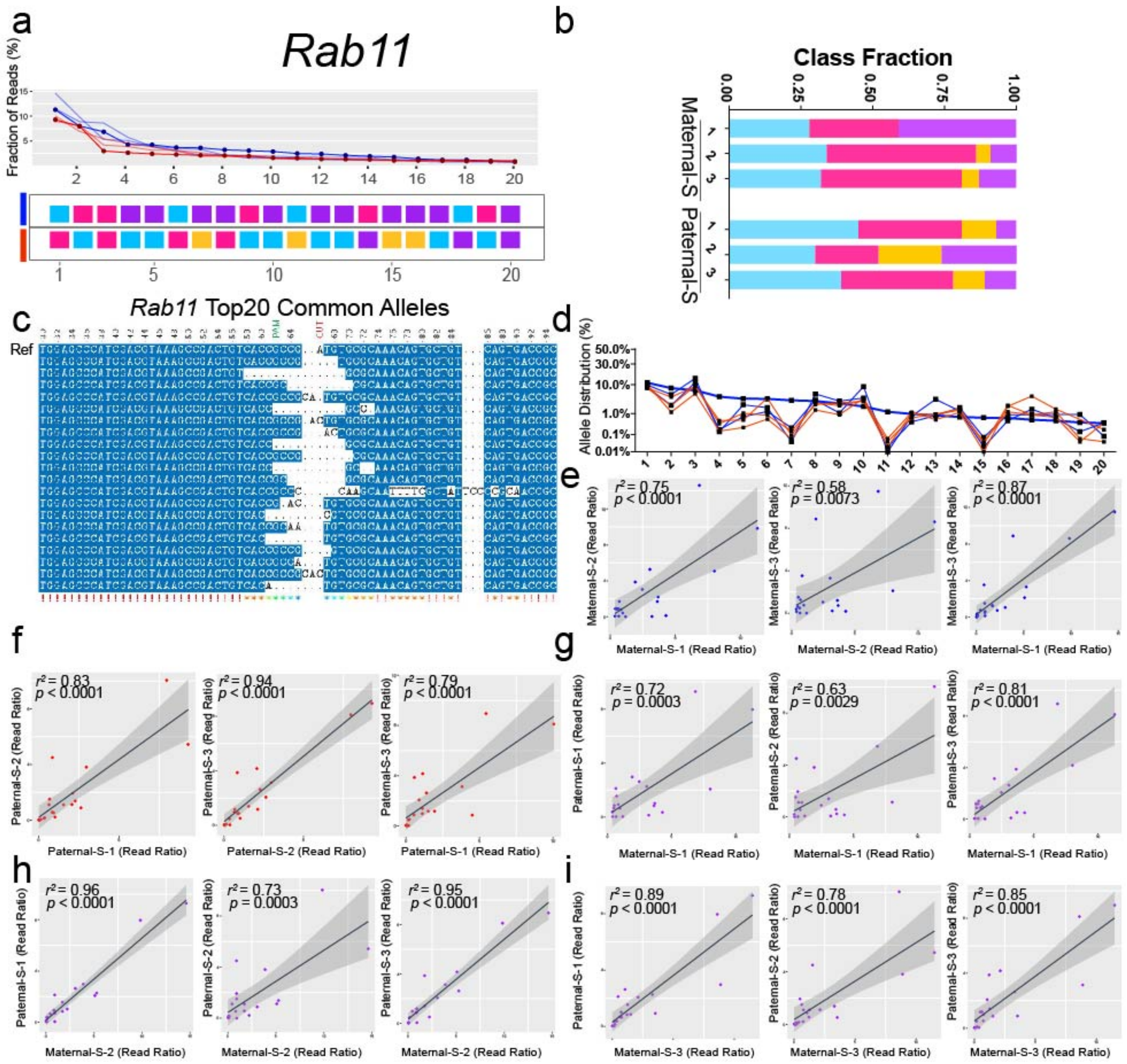

Suppl Figure 5. ICP analysis of somatic DSB repair outcomes generated by *Rab11* gRNA. a. Allele landscapes and classification fingerprints of top 20 alleles from *Rab11* Maternal-S (dark blue) and Paternal-S (red) crosses. For classification fingerprints, each allele is color coded with their categories. **b**. Class Fraction for top 50 alleles. **c**. Top 20 common alleles across all six sequenced individual flies aligned by multiple sequences alignment (MSA) method. The first row is the reference sequence. Blue letters show consensus among all aligned alleles. **d**. Allele landscapes of top 20 common alleles. X-axis shows alleles ranked by allele frequency, and the y-axis is the fraction of each allele among all reads, showing the probability. Dark blue lines are alleles generated from Maternal-S cross and red lines indicate Paternal-S cross. **e-i**. Correlation analysis either between two samples within the Maternal-S cross (e), Paternal-S (f), or different combinations between two crosses (g, h, i). Source data are provided as a Source Data file. r^2 values and p-values are indicated.

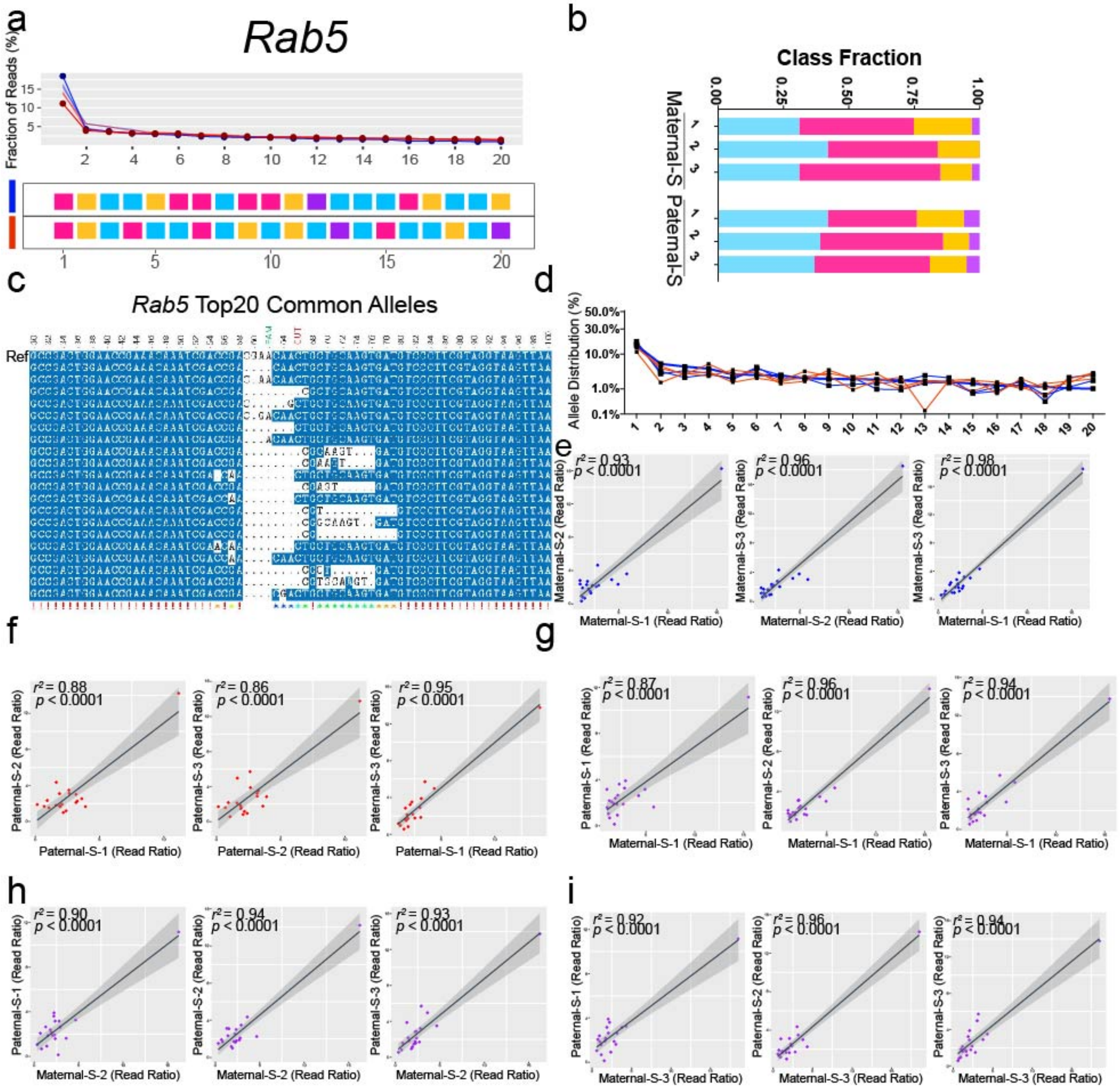

Suppl Figure 6. ICP analysis for somatic DSB repair outcomes generated by *Rab5* gRNA. a. Allele landscapes and classification fingerprints for top 20 alleles from *Rab5* Maternal-S (dark blue) and Paternal-S (red) crosses. For classification fingerprints, each allele is color coded with the categories. **b**. Class Fraction Index for top 50 alleles. **c**. Top 20 common alleles across all six sequenced individual flies aligned with multiple sequences alignment (MSA) method. The first row is the reference sequence. Blue letters show consensus among all aligned alleles. **d**. Allele landscapes of top 20 common alleles. X-axis is alleles ranked by frequency, and the y-axis is the fraction of each allele among all reads, showing the probability. Dark blue lines are alleles generated from Maternal-S cross and red lines indicate Paternal-S cross. **e-i**. Correlation analysis either between two samples within the Maternal-S cross (e),

Paternal-S (f), or different combinations between two crosses (g, h, i). Source data are provided as a Source Data file. r^2 values and p-values are indicated.

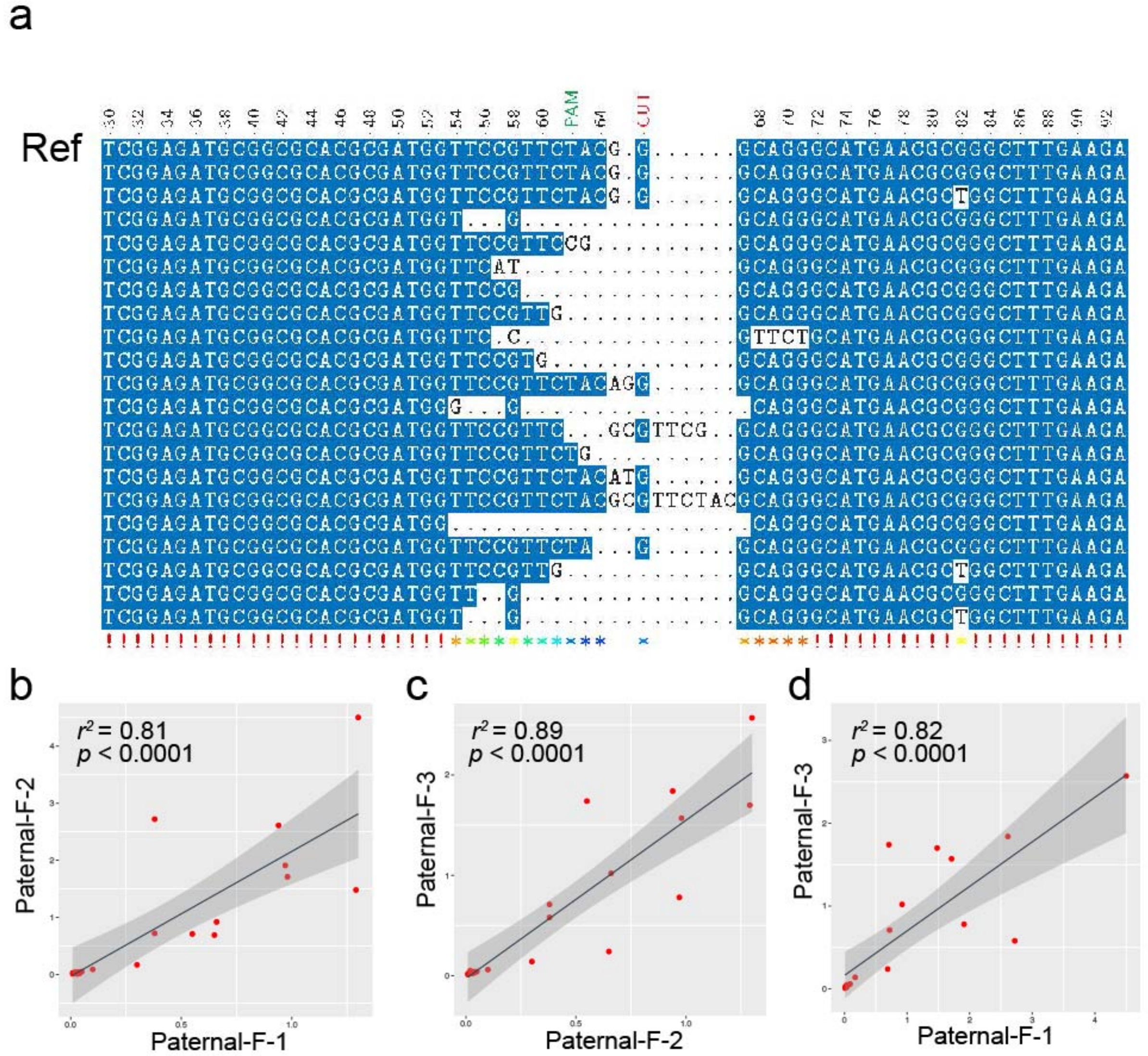

Suppl Figure 7. ICP analysis for Rec*kh* gRNA generated indels in somatic cells. a. MSA alignment of top 20 common alleles extracted from three individually sequenced F_1 mosquitoes of Paternal-F crosses. The first row shows the reference sequence, and blue letters show the consensus. **b-d**. Correlation analysis of NGS data generated from two out of three sequenced mosquitoes. r^2 values are listed. Source data are provided as a Source Data file. r^2 values and p-values are indicated.

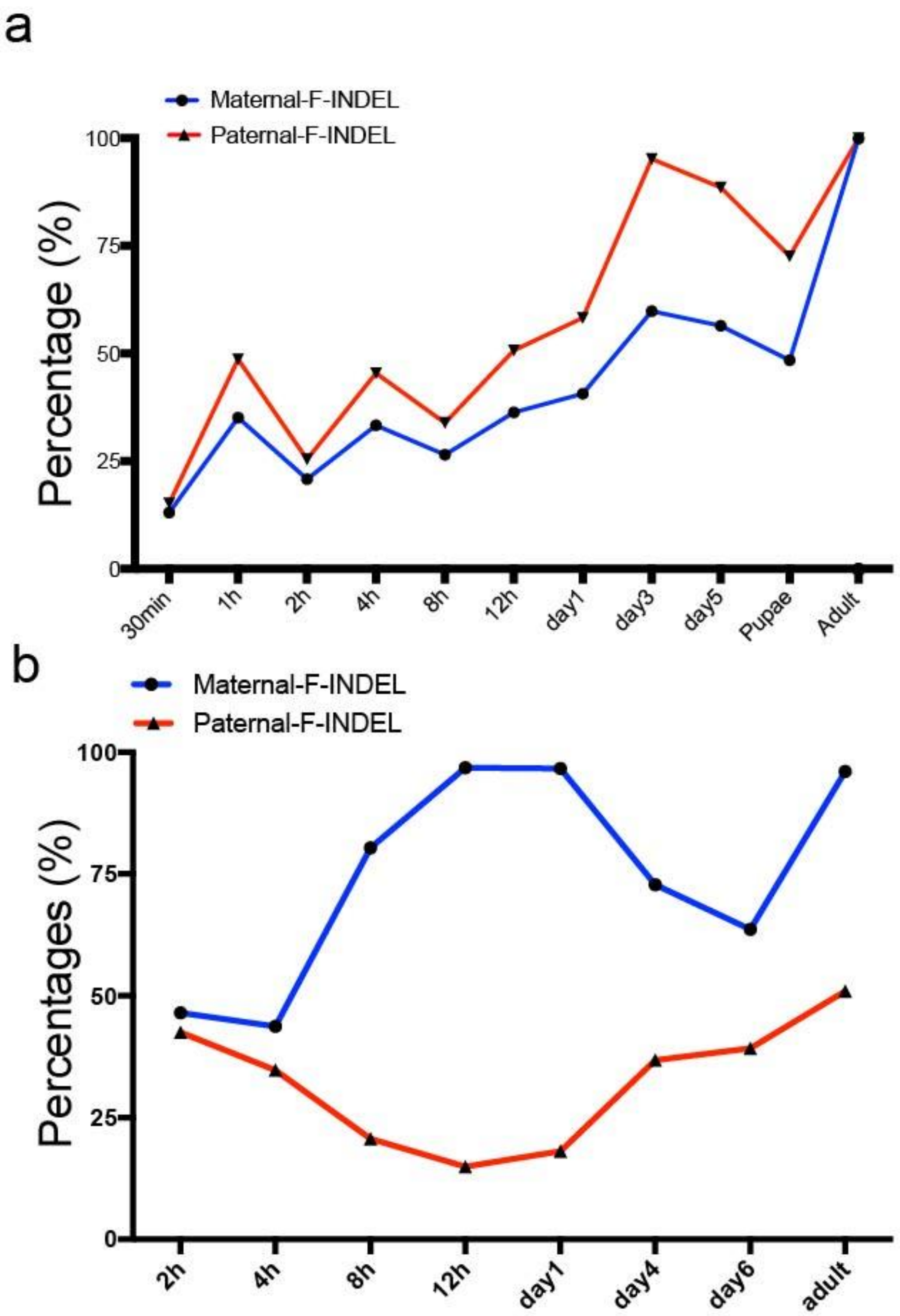

Suppl Figure 8. Kinetics of Cas9 mutagenesis generated by the *prosα2* (a) and Rec*kh* (b) gRNAs. Dark-blue lines indicate the fraction of Indels generated from maternal (Maternal-F) crosses and red lines are for paternal (Paternal-F) crosses. Source data are provided as a Source Data file.

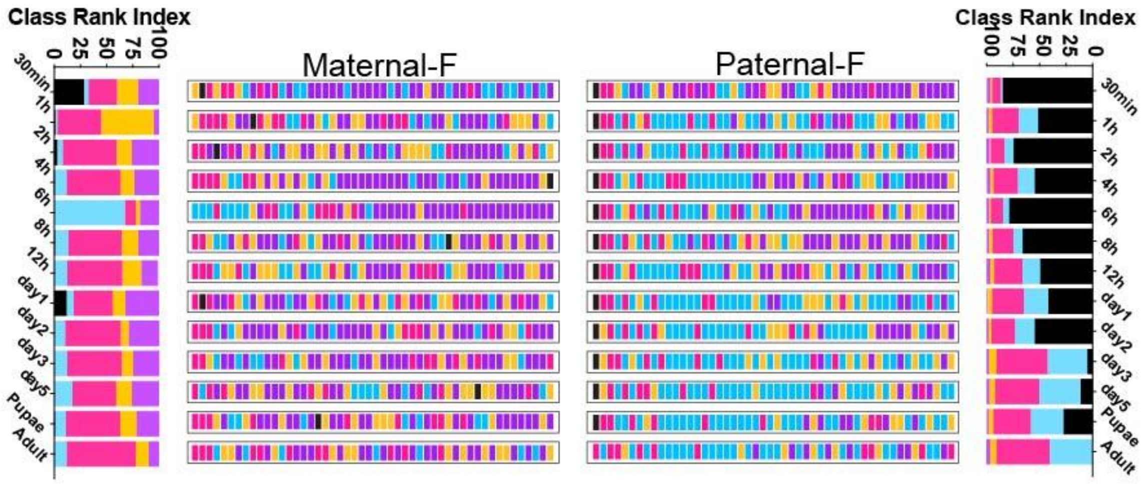

Suppl Figure 9. DSB repair fingerprints at different timepoints generated by *prosa2* gRNA in somatic cells. The far left and far right panels indicate the Class percentages including WT alleles (black), displaying the proportion of each class at single time-points. Source data are provided as a Source Data file.

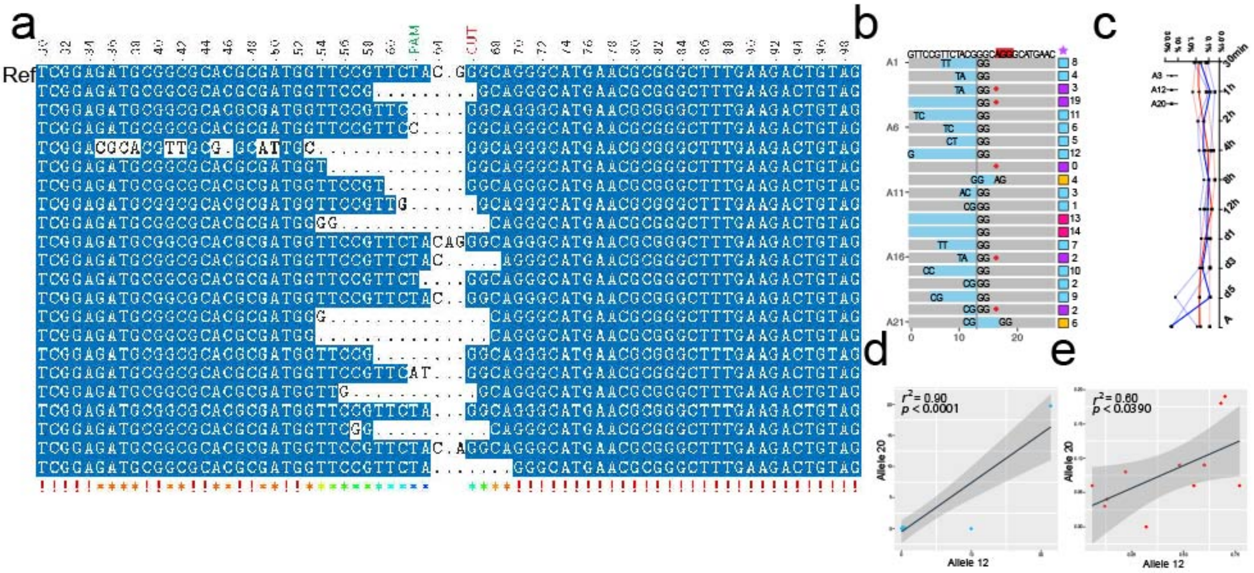

Suppl Figure 10. Correlation analysis for Rec*kh* gRNA generated somatic DSB repair outcomes with time-course assay a. MSA alignment of 21 common alleles extracted from all F_1 samples at different time-points with maternal and paternal lineage. **b**. Sequences of 21 common alleles. The first row shows the reference sequences centered by the expected DSB cleavage site. Red rectangle indicates the PAM sequence. On the right side, the color-coded squares indicate allele categories. **c**. Profiles of allele 3, 12 and 20 along with mosquito developmental process. Dark blue lines are Maternal-F crosses and red lines are Paternal-F crosses. Lines with dots stand for allele 3 (A3), lines with triangles are 12 (A12) and lines with rectangles are 20 (A20). **d, e**. Correlation analysis between

two alleles between allele 12 and 20. Source data are provided as a Source Data file. r^2 values and p-values are indicated.

Suppl Figure 11. Three generations cross schema for allele tracking with *ple*CC gRNA and Re*ckh* gRNA. a is for Maternal-F and Paternal-F crosse schemes and **b** is the illustration for next generation deep sequencing.

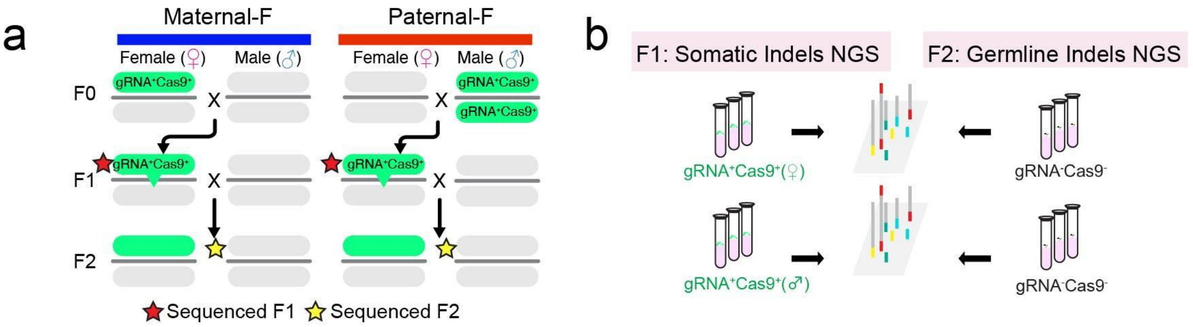

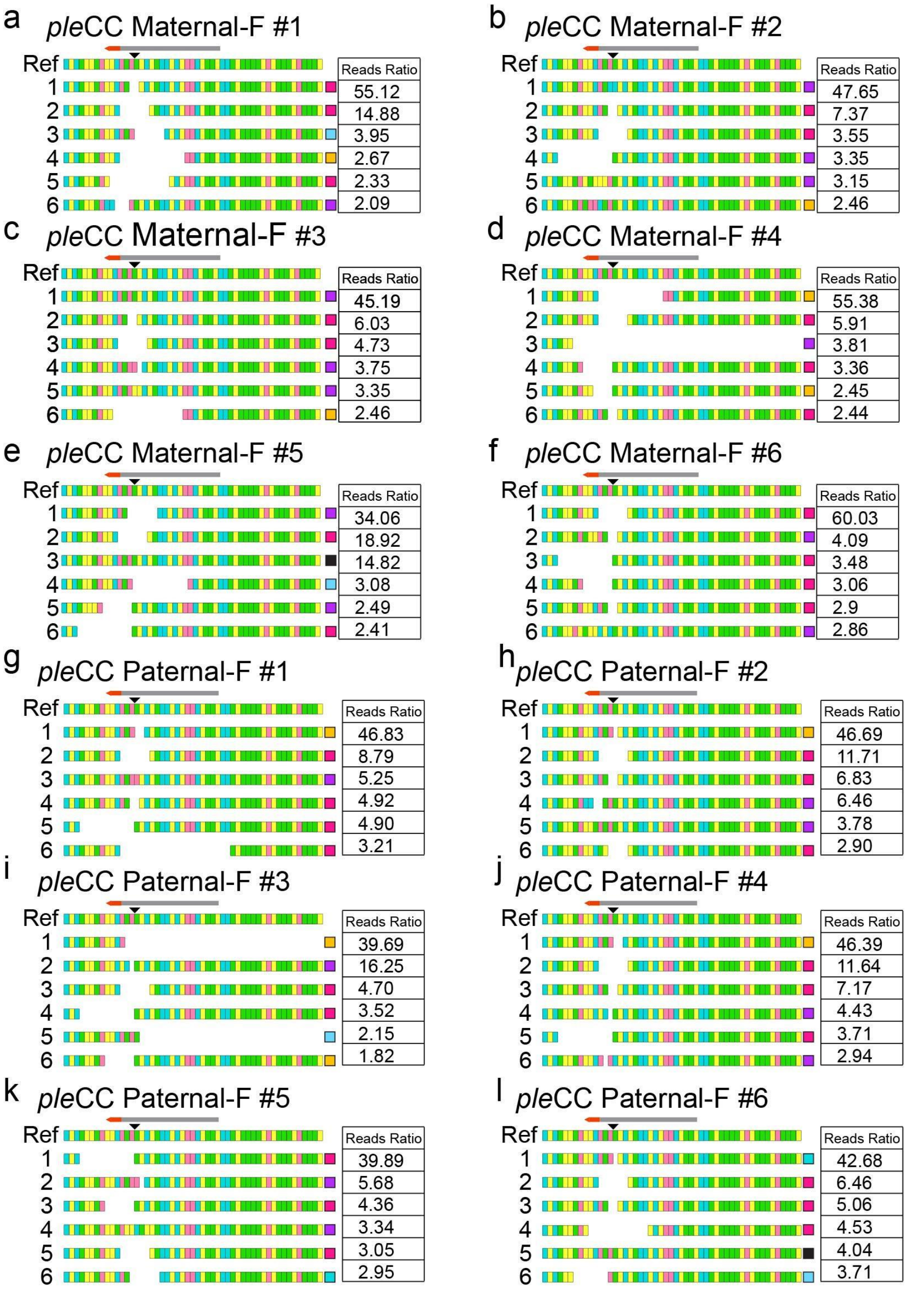

Suppl Figure 12. Top 6 germline alleles from F_2 progeny. Each panel shows DSB repair outcomes of individual F_2 progeny from crossing F_1 $Cas9^+gRNA^+$ females with wild-type males. **a-f.** Top 6 germline alleles of six F_2 test flies from F_0 Maternal-F crosses with *ple*CC gRNA and *vasa*-Cas9. **g-l**: Top 6 germline alleles of six F_2 test flies from F_0 Paternal-F crosses with *ple*CC gRNA and *vasa*-Cas9.

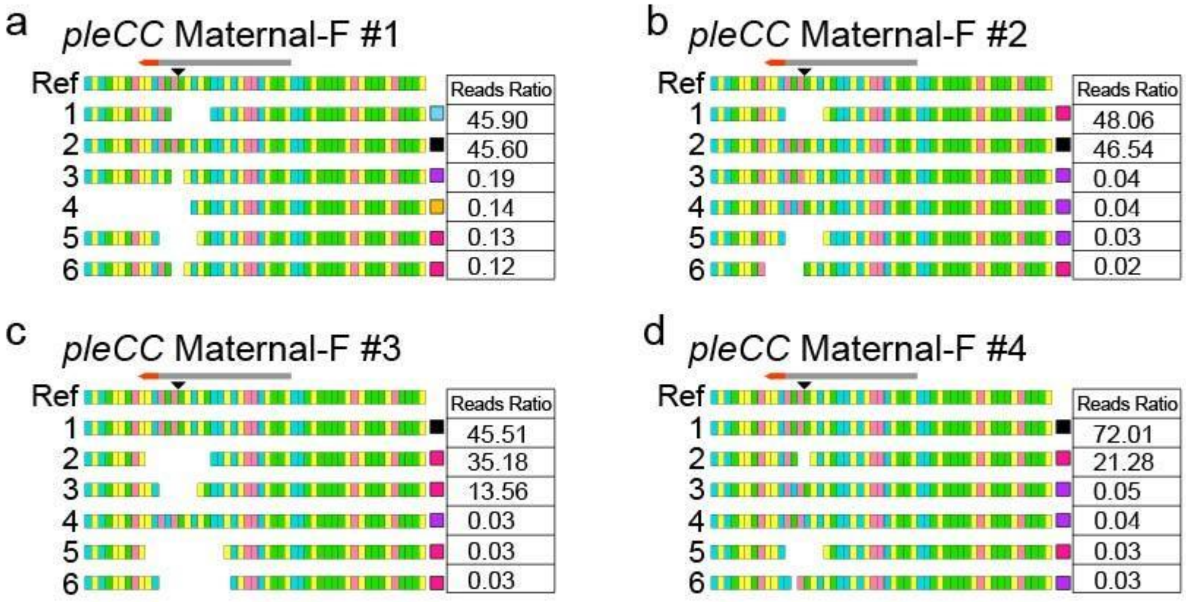

Suppl Figure 13. Top 6 germline alleles of F_2 progeny generated from F_1 Cas9$^+$gRNA$^+$ males. a-d shows top 6 germline alleles being generated in four individual F_2 progeny.
