# Supplementary material for: Developmental progression of DNA double-strand break repair deciphered by a single-allele resolution mutation classifier
Source: Nat Commun. 2024 Mar 23;15:2629. doi: 10.1038/s41467-024-46479-2 (PMC10960810; doi:10.1038/s41467-024-46479-2)
Supplement: Supplementary file 3 — Reporting Summary [file 41467_2024_46479_MOESM3_ESM.pdf]

nature portfolio

Corresponding author(s): Ethan Bier

Last updated by author(s): Jan 8, 2024

Reporting Summary

Nature Portfolio wishes to improve the reproducibility of the work that we publish. This form provides structure for consistency and transparency in reporting. For further information on Nature Portfolio policies, see our Editorial Policies and the Editorial Policy Checklist.

Statistics

For all statistical analyses, confirm that the following items are present in the figure legend, table legend, main text, or Methods section.

n/a	Confirmed	
☐	☒	The exact sample size (*n*) for each experimental group/condition, given as a discrete number and unit of measurement
☐	☒	A statement on whether measurements were taken from distinct samples or whether the same sample was measured repeatedly
☒	☐	The statistical test(s) used AND whether they are one- or two-sided<br>*Only common tests should be described solely by name; describe more complex techniques in the Methods section.*
☐	☒	A description of all covariates tested
☒	☐	A description of any assumptions or corrections, such as tests of normality and adjustment for multiple comparisons
☐	☒	A full description of the statistical parameters including central tendency (e.g. means) or other basic estimates (e.g. regression coefficient) AND variation (e.g. standard deviation) or associated estimates of uncertainty (e.g. confidence intervals)
☒	☐	For null hypothesis testing, the test statistic (e.g. *F*, *t*, *r*) with confidence intervals, effect sizes, degrees of freedom and *P* value noted<br>*Give P values as exact values whenever suitable.*
☒	☐	For Bayesian analysis, information on the choice of priors and Markov chain Monte Carlo settings
☐	☒	For hierarchical and complex designs, identification of the appropriate level for tests and full reporting of outcomes
☐	☒	Estimates of effect sizes (e.g. Cohen's *d*, Pearson's *r*), indicating how they were calculated

Our web collection on statistics for biologists contains articles on many of the points above.

Software and code

Policy information about availability of computer code

Data collection	Zeiss Stemi 2000 fluorescence microscope were used to acquire Drosophila thorax image, confocal images were acquired with Leica TCS SP8X confocal microscope. Microsoft Excel 2019 (v16.30) were used for data collection.
Data analysis	Images were analyzed with Leica Application Suite X. Fiji (OS version) and Photoshop (Photoshop CC v20.0.7) were used to adjust contrast and brightness of images, Helicon Focus (v7.6.1 Pro) was used to stack all images. GraphPad Prism 8 (v8.2.1) was used for data analysis and display. SnapGene (v5.0.7) was used for Sanger sequencing analysis. R studio (v4.1.0) was used for NGS data analysis.

For manuscripts utilizing custom algorithms or software that are central to the research but not yet described in published literature, software must be made available to editors and reviewers. We strongly encourage code deposition in a community repository (e.g. GitHub). See the Nature Portfolio guidelines for submitting code & software for further information.

Data

Policy information about availability of data

All manuscripts must include a data availability statement. This statement should provide the following information, where applicable:

- Accession codes, unique identifiers, or web links for publicly available datasets
- A description of any restrictions on data availability
- For clinical datasets or third party data, please ensure that the statement adheres to our policy

The sequences of hthCC plasmid used in this study have been deposited into GenBank Database under accession number OQ681082 [https://www.ncbi.nlm.nih.gov/nuccore/OQ681082]. All other plasmids refer to the publications Gerard et al., 2021 and Li et al., 2021. NGS raw sequencing data has been

deposited at the NCBI Sequence Read Archive database under Bioproject PRJNA978340 [https://www.ncbi.nlm.nih.gov/bioproject/?term=PRJNA978340], PRJNA978619 [https://www.ncbi.nlm.nih.gov/bioproject/?term=PRJNA978619], PRJNA979933 [https://www.ncbi.nlm.nih.gov/bioproject/?term=PRJNA979933], PRJNA979941 [https://www.ncbi.nlm.nih.gov/bioproject/?term=PRJNA979941], PRJNA980914 [https://www.ncbi.nlm.nih.gov/bioproject/?term=PRJNA980914], PRJNA980915 [https://www.ncbi.nlm.nih.gov/bioproject/?term=PRJNA980915], PRJNA981558 [https://www.ncbi.nlm.nih.gov/bioproject/?term=PRJNA981558]. Source data is provided in this paper as a Source Data File.

Research involving human participants, their data, or biological material

Policy information about studies with human participants or human data. See also policy information about sex, gender (identity/presentation), and sexual orientation and race, ethnicity and racism.

Reporting on sex and gender	This study has no experiment involves in human participants, their data or biological material.
Reporting on race, ethnicity, or other socially relevant groupings	Not applicable.
Population characteristics	Not applicable.
Recruitment	Not applicable.
Ethics oversight	Not applicable.

Note that full information on the approval of the study protocol must also be provided in the manuscript.

Field-specific reporting

Please select the one below that is the best fit for your research. If you are not sure, read the appropriate sections before making your selection.

☒ Life sciences ☐ Behavioural & social sciences ☐ Ecological, evolutionary & environmental sciences

For a reference copy of the document with all sections, see nature.com/documents/nr-reporting-summary-flat.pdf

Life sciences study design

All studies must disclose on these points even when the disclosure is negative.

Sample size	Our previous experience of similar analysis for somatic DSB repair outcomes suggested sequencing 20 individual flies is usually representative for bulk deep sequencing (Li et al., 2021). For single fly or mosquito somatic DSB repair analysis, at least 3 individual animals were used for sequencing and data analysis.
Data exclusions	Fly crosses with no progeny due to contamination or other causes were removed from the analysis. Other data were not excluded from analysis.
Replication	For single fly or mosquito deep sequencing, at least 3 biological replications were conducted. Samples failed with sequencing either because of poor library preparation or sequencing issue were discarded from the data. At least 3 cages were run at the same time for cage trials. Other replication details are addressed in the text.
Randomization	20 F1 progeny were randomly selected and pooled together for deep sequencing. F1 females or males were randomly collected from different F0 crosses to perform F1 crosses.
Blinding	In all fly experiments performed we had checked fluorescence presence in the eyes of fruit flies. This type of scoring does not need the investigators to be blind as the evaluation of the phenotype is presence or absence of the fluorescence marker and there is no much room for interpretation that could be subjective.

Reporting for specific materials, systems and methods

We require information from authors about some types of materials, experimental systems and methods used in many studies. Here, indicate whether each material, system or method listed is relevant to your study. If you are not sure if a list item applies to your research, read the appropriate section before selecting a response.

Materials & experimental systems

n/a	Involved in the study
☐	☒ Antibodies
☒	☐ Eukaryotic cell lines
☒	☐ Palaeontology and archaeology
☐	☒ Animals and other organisms
☒	☐ Clinical data
☒	☐ Dual use research of concern
☒	☐ Plants

Methods

n/a	Involved in the study
☒	☐ ChIP-seq
☒	☐ Flow cytometry
☒	☐ MRI-based neuroimaging

Antibodies

Antibodies used	Sheep anti-Dig 488, mouse anti-Bio555, rabbit anti-FITC.
Validation	All antibodies have been validated by the vendors and ourselves for in situ FISH (https://www.science.org/doi/10.1126/science.1099247?url_ver=Z39.88-2003&rfr_id=ori:rid:crossref.org&rfr_dat=cr_pub%20%200pubmed).

Animals and other research organisms

Policy information about studies involving animals; ARRIVE guidelines recommended for reporting animal research, and Sex and Gender in Research

Laboratory animals	Transgenic flies being generated from wild-type W118 and Oregon-R flies. Reporting on age is not applicable for this study. Calf blood (Colorado Serum Co., Denver) was used for mosquitoes blood feeding.
Wild animals	No wild animals were used in this study.
Reporting on sex	Reporting on sex is not applicable for this study.
Field-collected samples	No field-collected samples were used in this study.
Ethics oversight	Drosophila melonogaster is an invertebrate and is exempt from IACUC oversight.

Note that full information on the approval of the study protocol must also be provided in the manuscript.

Plants

Seed stocks	Not applicable.
Novel plant genotypes	Not applicable.
Authentication	Not applicable.
